# Trends in Targeted Therapy Usage in Inflammatory Bowel Disease: TRENDY Study of ENEIDA

**DOI:** 10.3390/pharmaceutics16050629

**Published:** 2024-05-08

**Authors:** Celia Gómez-Labrador, Elena Ricart, Marisa Iborra, Eva Iglesias, María Dolores Martín-Arranz, Luisa de Castro, Ruth De Francisco, Francisco Javier García-Alonso, Ana Sanahuja, Carla J. Gargallo-Puyuelo, Francisco Mesonero, María José Casanova, Míriam Mañosa, Montserrat Rivero, Marta Calvo, Mónica Sierra-Ausin, Carlos González-Muñoza, Xavier Calvet, Santiago García-López, Jordi Guardiola, Lara Arias García, Lucía Márquez-Mosquera, Ana Gutiérrez, Yamile Zabana, Mercè Navarro-Llavat, Rufo Lorente Poyatos, Marta Piqueras, Leyanira Torrealba, Fernando Bermejo, Ángel Ponferrada-Díaz, José L. Pérez-Calle, Manuel Barreiro-de Acosta, Coral Tejido, José Luis Cabriada, Ignacio Marín-Jiménez, Óscar Roncero, Yolanda Ber, Luis Fernández-Salazar, Blau Camps Aler, Alfredo J. Lucendo, Jordina Llaó, Luis Bujanda, Carmen Muñoz Villafranca, Eugeni Domènech, María Chaparro, Javier P. Gisbert

**Affiliations:** 1 Gastroenterology Unit, Hospital Universitario de La Princesa, Instituto de Investigación Sanitaria Princesa (IIS-Princesa), Universidad Autónoma de Madrid and Centro de Investigación Biomédica en Red de Enfermedades Hepáticas y Digestivas (CIBEREHD), 28006 Madrid, Spain; mjcasanova.g@gmail.com (M.J.C.); mariachs2005@gmail.com (M.C.); javier.p.gisbert@gmail.com (J.P.G.); 2 Gastroenterology Unit, Hospital Clinic of Barcelona, Institut d’Investigacions Biomèdiques August Pi i Sunyer (IDIBAPS) and CIBEREHD, 08036 Barcelona, Spain; 3 Gastroenterology Unit, Hospital Universitario y Politécnico La Fe, 46026 Valencia, Spain; marisaiborra@hotmail.com; 4 Gastroenterology Unit, Hospital Universitario Reina Sofía, Instituto Maimónides de Investigación Biomédica de Córdoba (IMIBIC), 14004 Córdoba, Spain; evaiflores@gmail.com; 5 Gastroenterology Unit, Hospital Universitario La Paz, Universidad Autónoma de Madrid, Hospital La Paz Institute for Health Research, 28046 Madrid, Spain; martinarranz.lapaz@gmail.com; 6 Gastroenterology Unit, Hospital Álvaro Cunqueiro, Grupo de Investigación en Patología Digestiva, Instituto de Investigación Sanitaria Galicia Sur (IIS Galicia Sur) SERGAS, UVIGO, 36312 Vigo, Spain; maria.luisa.de.castro.parga@sergas.es; 7 Gastroenterology Unit, Hospital Universitario Central de Asturias and Instituto de Investigación Sanitaria del Principado de Asturias (ISPA), 33011 Oviedo, Spain; ruthdefrancisco@gmail.com; 8 Gastroenterology Unit, Hospital Universitario Río Hortega, 47012 Valladolid, Spain; fj.garcia.alonso@gmail.com; 9 Gastroenterology Unit, Hospital Clínico Universitario de Valencia, 46010 Valencia, Spain; anasanahujamar@gmail.com; 10 Gastroenterology Unit, Hospital Clínico Universitario Lozano Blesa, IIS Aragón, 50009 Zaragoza, Spain; carlajerusalen@hotmail.com; 11 Gastroenterology Unit, Hospital Universitario Ramón y Cajal, 28034 Madrid, Spain; pacomeso@hotmail.com; 12 Gastroenterology Unit, Hospital Universitari Germans Trias i Pujol, and CIBEREHD, 08916 Badalona, Spain; mmanosa.germanstrias@gencat.cat (M.M.); eugenidomenech@gmail.com (E.D.); 13 Gastroenterology Unit, Hospital Universitario de Valdecilla and Instituto de Investigación Sanitaria Valdecilla (IDIVAL), 39008 Santander, Spain; montserrat.rivero@scsalud.es; 14 Gastroenterology Unit, Hospital Universitario Puerta de Hierro Majadahonda, 28222 Madrid, Spain; calvo.marta@gmail.com; 15 Gastroenterology Unit, Complejo Asistencial Universitario de León, 24008 León, Spain; msierra.ausin@gmail.com; 16 Gastroenterology Unit, Hospital de la Santa Creu i Sant Pau, 08025 Barcelona, Spain; cgonzalezm@santpau.cat; 17 Gastroenterology Unit, Parc Taulí, Hospital Universitari, Institut d’Investigació i Innovació Parc Taulí, Departament de Medicina, Universitat Autònoma de Barcelona and CIBEREHD, 08208 Sabadell, Spain; xcalvet@tauli.cat; 18 Gastroenterology Unit, Hospital Universitario Miguel Servet, e Instituto de Investigación Sanitaria de Aragón (IIS Aragón), 50009 Zaragoza, Spain; sgarcia.lopez@gmail.com; 19 Gastroenterology Unit, Hospital Universitario de Bellvitge, Instituto de Investigación Biomédica de Bellvitge (IDIBELL), Universitat de Barcelona, 08907 Barcelona, Spain; jguardiola@bellvitgehospital.cat; 20 Gastroenterology Unit, Hospital Universitario de Burgos, 09006 Burgos, Spain; laradigest@yahoo.es; 21 Gastroenterology Unit, Hospital del Mar, Barcelona and IMIM (Hospital del Mar Medical Research Institute), 08003 Barcelona, Spain; lmarquez@parcdesalutmar.cat; 22 Gastroenterology Unit, Hospital General Universitario de Alicante, CIBEREHD, Instituto de Investigación Sanitaria y Biomédica de Alicante (ISABIAL), 03010 Alicante, Spain; gutierrez_anacas@gva.es; 23 Gastroenterology Unit, Hospital Universitario Mútua Terrassa, and CIBEREHD, 08221 Terrassa, Spain; yzabana@gmail.com; 24 Gastroenterology Unit, Complex Hospitalari Universitari Moisès Broggi, 08970 Barcelona, Spain; merce.navarro@sanitatintegral.org; 25 Gastroenterology Unit, Hospital General Universitario de Ciudad Real, 13005 Ciudad Real, Spain; rufolorente@hotmail.com; 26 Gastroenterology Unit, Consorci Sanitari Terrassa, 08227 Terrassa, Spain; piqueras72@gmail.com; 27 Gastroenterology Unit, Hospital Universitario Dr. Josep Trueta, 17007 Girona, Spain; leyatorrealba@gmail.com; 28 Gastroenterology Unit, Hospital Universitario de Fuenlabrada, 28942 Madrid, Spain; fernando.bermejo@salud.madrid.org; 29 Gastroenterology Unit, Hospital Universitario Infanta Leonor, 28031 Madrid, Spain; angelponmedicina@yahoo.es; 30 Gastroenterology Unit, Hospital Universitario Fundación Alcorcón, 28922 Madrid, Spain; jlperezc@fhalcorcon.es; 31 Gastroenterology Unit, Hospital Clínico Universitario de Santiago de Compostela, 15706 Santiago de Compostela, Spain; manubarreiro@hotmail.com; 32 Gastroenterology Unit, Complexo Hospitalario Universitario de Ourense, 32005 Ourense, Spain; coral.tejido.sandoval@sergas.es; 33 Gastroenterology Unit, Hospital de Galdakao-Usansolo, 48960 Galdakao, Spain; jcabriada@gmail.com; 34 Gastroenterology Unit, Hospital Gregorio Marañón, Instituto de Investigación Sanitaria Gregorio Marañón (IiSGM), Universidad Complutense de Madrid, 28007 Madrid, Spain; drnachomarin@hotmail.com; 35 Gastroenterology Unit, Complejo Hospitalario la Mancha Centro, 13600 Alcázar de San Juan, Spain; dr.roncero@gmail.com; 36 Gastroenterology Unit, Hospital Universitario San Jorge, 22004 Huesca, Spain; ybernieto@gmail.com; 37 Gastroenterology Unit, Hospital Clínico Universitario de Valladolid (SACYL), Universidad de Valladolid, 47003 Valladolid, Spain; luisfernsal@gmail.com; 38 Gastroenterology Unit, Hospital General de Granollers, 08402 Granollers, Spain; blaucamps@gmail.com; 39 Gastroenterology Unit, Hospital General de Tomelloso, Instituto de Investigación Sanitaria de Castilla-La Mancha (IDISCAM), and CIBEREHD, 13700 Tomelloso, Spain; ajlucendo@hotmail.com; 40 Gastroenterology Unit, Althaia Xarxa Assistencial Universitaria de Manresa, 08243 Barcelona, Spain; jllao@althaia.cat; 41 Gastroenterology Unit, Hospital Universitario Donostia, Instituto Biodonostia, CIBEREHD and Universidad del País Vasco (UPV/EHU), 20014 San Sebastián, Spain; luis.bujanda@osakidetza.net; 42 Gastroenterology Unit, Hospital de Basurto, 48013 Bilbao, Spain; mariadelcarmen.munozvillafranca@osakidetza.net; 43 Universitat Autònoma de Barcelona, 08193 Barcelona, Spain

**Keywords:** biologics, biosimilars, targeted therapy, inflammatory bowel disease, Crohn’s disease, ulcerative colitis, trends, positioning

## Abstract

Markers that allow for the selection of tailored treatments for individual patients with inflammatory bowel diseases (IBD) are yet to be identified. Our aim was to describe trends in real-life treatment usage. For this purpose, patients from the ENEIDA registry who received their first targeted IBD treatment (biologics or tofacitinib) between 2015 and 2021 were included. A subsequent analysis with Machine Learning models was performed. The study included 10,009 patients [71% with Crohn’s disease (CD) and 29% with ulcerative colitis (UC)]. In CD, anti-TNF (predominantly adalimumab) were the main agents in the 1st line of treatment (LoT), although their use declined over time. In UC, anti-TNF (mainly infliximab) use was predominant in 1st LoT, remaining stable over time. Ustekinumab and vedolizumab were the most prescribed drugs in 2nd and 3rd LoT in CD and UC, respectively. Overall, the use of biosimilars increased over time. Machine Learning failed to identify a model capable of predicting treatment patterns. In conclusion, drug positioning is different in CD and UC. Anti-TNF were the most used drugs in IBD 1st LoT, being adalimumab predominant in CD and infliximab in UC. Ustekinumab and vedolizumab have gained importance in CD and UC, respectively. The approval of biosimilars had a significant impact on treatment.

## 1. Introduction

Inflammatory bowel disease (IBD) encompasses two conditions, Crohn’s disease (CD) and ulcerative colitis (UC), that are chronic, relapsing disorders with an increasing incidence and prevalence worldwide. Overall, approximately 1.3 million people in Europe suffer from IBD, which mainly affects the young population [1]. IBD is a very disabling condition due to its association with significant morbidity caused by hospitalizations and surgeries [2]. Since currently there is no curative treatment, the therapeutic objective is controlling the inflammatory process to prevent relapses and complications [3]. IBD is a very relevant disease with a high social burden due to its complexity and the costs (direct and indirect) associated with its treatment [3]. Before the approval of biological agents, surgery and hospitalization were the main cost drivers in IBD, which accounted for more than half of the total cost. In recent years, IBD treatment has evolved to rely increasingly on medical treatment, especially biologics; therefore, healthcare costs have shifted from hospitalization and surgery to biologics-related expenses [4].

Increasing knowledge of the role of pro-inflammatory cytokines and immune cell components in the pathogenesis of the disease has led to the approval in recent decades of targeted therapies that have revolutionized IBD management [5]. These therapies, based on biological drugs include anti-TNF [monoclonal antibodies (mAb) targeting TNF; infliximab, adalimumab and golimumab], vedolizumab (mAb against α4β7 integrin), ustekinumab (mAb against the p40 subunit shared by interleukins 12 and 23), and more recently, JAK inhibitors (small molecules inhibiting JAK-STAT signaling pathways; tofacitinib, filgotinib and upadacitinib) [6]. Moreover, many other biologics are expected to be available in the future. Although these drugs are effective in inducing and maintaining remission, primary and secondary non-response rates remain high. Furthermore, direct comparisons of their efficacy are uncommon in the literature and most of this information is based on indirect comparisons, such as network meta-analyses [2]. In the first published head-to-head trial, the VARSITY study, vedolizumab was superior to adalimumab achieving clinical remission and endoscopic improvement in bio-naïve UC patients, but not corticosteroid-free clinical remission [7]. However, the SEAVUE study found similar efficacy for ustekinumab and adalimumab in bio-naïve CD patients [8].

Currently, there is a lack of evidence supporting strategies for positioning targeted therapies in first and subsequent lines of IBD treatment. Anti-TNF agents have been the cornerstone of IBD therapy in recent decades and, mainly after the approval of biosimilars, they have been commonly used as the first targeted therapy in most patients (mainly due to reimbursement policies). However, approximately one-third of patients do not respond to anti-TNF induction therapy and up to 40% of patients may lose response in the first year of therapy [9]. To date, there is insufficient evidence to recommend specific second-line therapies after anti-TNF failure. The choice between a second anti-TNF drug and other therapies with a different mechanism of action (MoA) is often a decision based on clinician’s experience, drug availability or economic issues [10]. The complexity of the pathophysiological mechanisms of IBD, the heterogeneity of the disease and the great variability in the clinical response observed in patients has led to the development of new therapeutic targets and the search for predictive models to enable personalization of treatments [11,12]. In addition, new advances in drug delivery, such as the nano-delivery system, have been developed with the aim of improving efficacy and reducing the adverse effects of targeted therapies [13].

Since the therapeutic armamentarium for IBD has increased in recent years, characterizing the usage patterns of targeted therapies to date would help to guide the clinician in choosing the most appropriate treatment for each case. Thus, the aim of the present study was to describe the positioning trends of targeted therapies for IBD since 2015 (when the first non-anti-TNF biological agent became available in Spain), and to identify factors (related to IBD or external) that may influence the choice between different options.

## 2. Materials and Methods

### 2.1. Study Design and Population

This is a trend study, a type of epidemiological study that aims to describe how drug use patterns have evolved in a specific population over time. It is an observational, retrospective, multicenter study using data from the ENEIDA registry [14], which is a prospectively maintained registry created in 2005 and promoted by the Spanish Working Group on Crohn’s Disease and Ulcerative Colitis (GETECCU). Patients above 18 years of age included in the ENEIDA registry who started their first targeted treatment (biologics or tofacitinib) for IBD between 2015 (when the first non-anti-TNF biologic agent became available in Spain) and 2021 were analyzed. Only centers with high quality data and with at least 75% of their patients included in ENEIDA were considered in this study. The ENEIDA registry was approved by research ethics committees in all participating centers, and patients gave their written informed consent to be included in the registry.

Patients were followed from the starting date of the first targeted therapy to the last visit or end of follow-up, whichever came first. Patients who received the targeted drug for an indication different from IBD (i.e., extraintestinal manifestations) were excluded. The use of drugs was described based on their MoA and then, within each therapeutic group, including originators and biosimilars.

### 2.2. Data Collection

Only available data, obtained as part of the patient’s regular care, were collected. The following variables were considered: sociodemographic variables [sex, age at the date of first targeted therapy, hospital category (see below), and some known risk factors as family history of IBD and smoking habit] and clinical variables (IBD type, extension in UC, location and phenotype in CD according to Montreal classification, and presence of extraintestinal manifestations). For each targeted therapy (including biologics and tofacitinib), we analyzed the following variables: starting date, drug type (including whether the drug was originator or biosimilar), treatment indication, end date and reason for stopping the treatment. Immunomodulatory treatment use and the need for surgery were also registered. Healthcare centers were categorized in five levels depending on their complexity according to the IASIST classification (see Section 2.3). Efficacy and safety rates of targeted drugs were not evaluated in this study.

### 2.3. Definitions

#### 2.3.1. Targeted Therapies and Immunomodulatory Treatments

All targeted therapies already approved by the European Medicines Agency (EMA) during the study period were included; infliximab, adalimumab (both including all available biosimilars), ustekinumab and vedolizumab for CD; and these drugs, golimumab and tofacitinib for UC (other JAK inhibitors were not approved by the EMA at the time of data extraction). Immunomodulatory treatments included concomitant or prior treatment with ciclosporin, azathioprine, mercaptopurine or methotrexate.

#### 2.3.2. Change of Line

It was defined as the replacement of one targeted therapy by another, excluding the change from an originator drug to its biosimilar. The reasons for starting treatment (induction of remission, maintenance of remission, fistulising disease or prophylaxis of post-surgical recurrence) and those for stopping treatment [primary non-response (PNR), secondary loss of response (LOR), sustained remission or onset of adverse events] with the different drugs were those considered in ENEIDA; no special criteria were applied in this study.

#### 2.3.3. IASIST Score

The IASIST score was used to classify all healthcare centers in five categories according to their complexity (higher category meaning higher complexity), based on data from the National Catalogue of Hospitals [15]. Complexity was assessed depending on the number of beds, technological resources, and teaching accreditation. Hospitals within categories 1–3 were considered as low complexity hospitals, and those within categories 4–5 as high complexity hospitals.

### 2.4. Statistical Analysis

Initially, a descriptive analysis was performed. Categorical variables were expressed as frequencies and their related percentages, with 95% confidence intervals (95% CI). Quantitative variables were expressed as number of subjects, with means and standard deviations (SD) or medians and interquartile ranges (IQR), depending on whether they were normally distributed or not. Missing observations were detected and presented for each variable. In the univariate analysis, categorical variables were compared using the chi-square test, and a Cochran–Armitage trend test was performed to assess linear variation over time in treatment prescription. A *p*-value < 0.05 was considered statistically significant.

In a subsequent analysis, we evaluated potential factors influencing the selection of a particular drug. For this analysis, we used Machine Learning algorithms (XGBoost, Logistic Regression, Random Forest and Light Gradient Boosting Machine) to find a model capable of predicting the targeted therapies prescribed to a patient depending on certain variables (sociodemographic, clinical, treatment and hospital-related variables). Several model iterations were run for the prediction of different targeted therapies in 1st and 2nd line of treatment (LoT) in CD and UC. In those models with better performance (determined by precision and recall), the model was interpreted to find possible patterns in the prescription of these therapies as well as the most relevant variables affecting them.

## 3. Results

### 3.1. Baseline Characteristics

**Figure 1:**
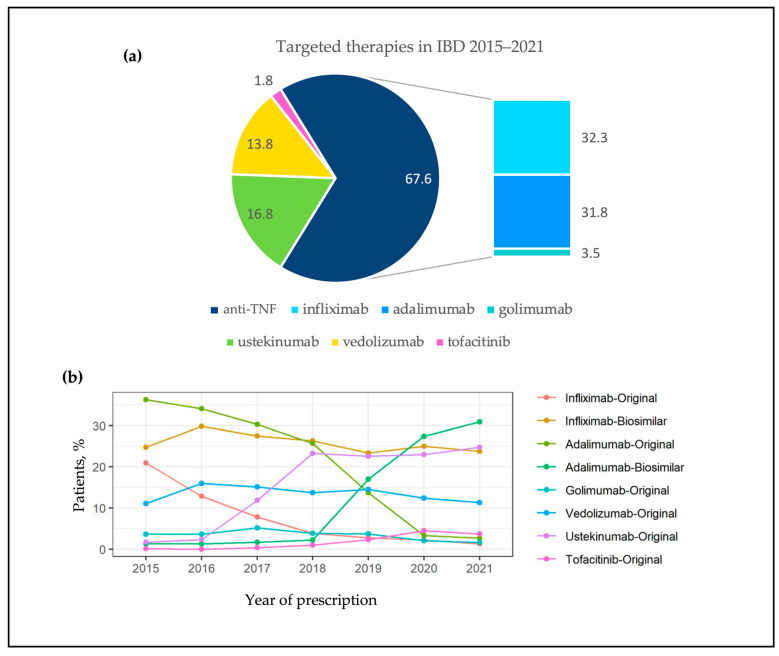
(**a**) Distribution of the use of targeted therapies for inflammatory bowel disease in Spain from 2015 to 2021. Values are provided as %. Inflammatory bowel disease (IBD); (**b**) evolution of targeted therapies prescription for inflammatory bowel disease (including originator and biosimilars) throughout the study period in Spain.

Between 2015 and 2021, a total of 10,009 patients with IBD from 42 Spanish centers were treated with a targeted therapy for the first time: 7089 (71%) had CD and 2920 (29%) UC. Patients’ sociodemographic and baseline characteristics are summarized in Table 1. Median age at the start time of the first targeted IBD therapy was 44 years, and 52.3% of the patients were male. Among patients with CD, ileocolic location (45%) and inflammatory behavior (55%) were the most common clinical characteristics, and 30% of patients had associated perianal disease. In UC, 54% of the patients exhibited extensive colitis. Twenty-seven percent of the patients with IBD presented extraintestinal manifestations. In total, 44% percent and 16% of CD and UC patients, respectively, were smokers at the time of diagnosis, and 16% of patients had a family history of IBD. Most patients with IBD were treated in high complexity hospitals (87%), although hospital complexity did not influence the choice of drug by MoA in both CD and UC (Supplementary Table S1). The majority of patients with IBD in our registry (68%) required only one LoT for disease control, and 35% of patients with IBD required at least one surgery (abdominal and/or perianal). Overall, anti-TNF were the most used drugs in IBD, followed by ustekinumab and vedolizumab (68%, 17% and 14%, respectively). Among anti-TNF, infliximab and adalimumab were similarly used (32%) (Figure 1a). However, the approval of new targeted therapies during the study period was associated with a relative decrease in the use of anti-TNF over time (87% in 2015 vs. 60% in 2021, *p* < 0.001) concomitant with an increase in ustekinumab and tofacitinib (Figure 1b, Supplementary Table S2).

### 3.2. Crohn’s Disease

#### 3.2.1. Patterns of Targeted Therapies by Line of Treatment

**Figure 2:**
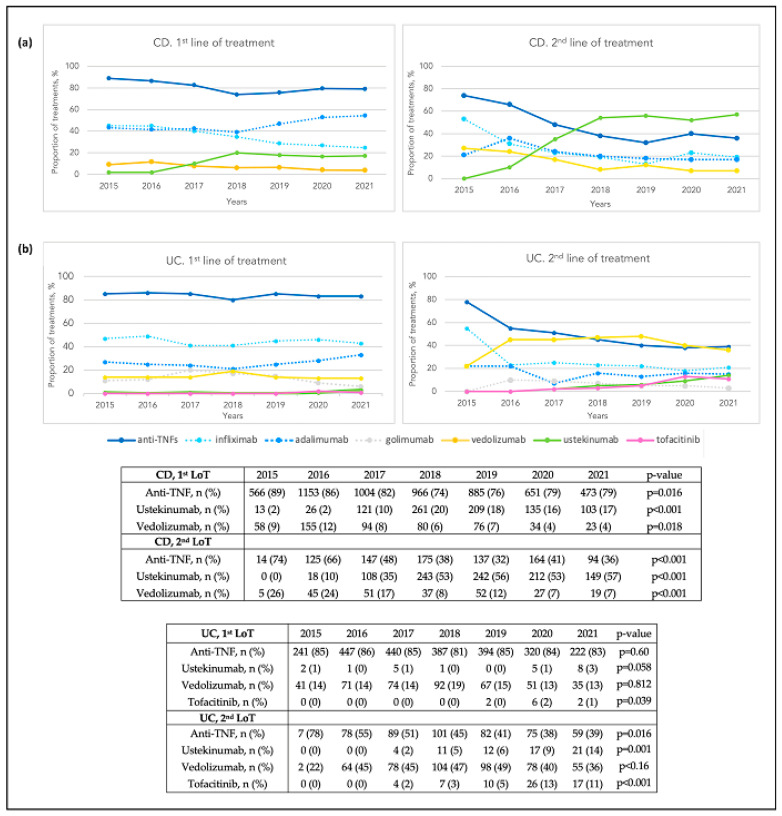
Prescription of targeted therapies used in Crohn’s disease (**a**) and in ulcerative colitis (**b**) per year in 1st and 2nd LoT. Crohn’s disease (CD); ulcerative colitis (UC); line of treatment (LoT).

In luminal CD, anti-TNF were the most used first-line agents, with a slight decline of their use over time in 1st LoT (89% in 2015 vs. 79% in 2021, *p* = 0.016) (Figure 2a). Among anti-TNF, adalimumab was more used in 1st LoT than infliximab throughout the entire study period (45% vs. 36%, *p* < 0.001) (Table 2A); furthermore, its use increased in recent years (43% in 2015 vs. 54% in 2021, *p* = 0.030). Until 2016, vedolizumab was the second most prescribed drug by MoA, followed by ustekinumab (12% vs. 2% in 2016, *p* < 0.001); however, since 2017 ustekinumab use progressively increased (10% in 2017 vs. 17% in 2021, *p* < 0.001), becoming the second most prescribed drug (17% ustekinumab vs. 4% vedolizumab in 2021, *p* < 0.001) (Figure 2a).

In 2nd LoT, anti-TNF were initially the most prescribed agents by MoA, but its use decreased during the study period (74% in 2015 vs. 36% in 2021, *p* < 0.001). Conversely, there was an increase in the use of ustekinumab (10% in 2016 vs. 57% in 2021, *p* < 0.001). Since 2018, ustekinumab became the most frequently used drug in 2nd LoT by MoA, followed by anti-TNF and vedolizumab (57% vs. 36% vs. 7% in 2021, *p* < 0.001) (Figure 2a).

In 3rd LoT, ustekinumab was the most prescribed drug by MoA throughout the whole study period, followed by anti-TNF and vedolizumab (53% vs. 26% vs. 20%, *p* < 0.001, respectively). In 4th LoT, and subsequent lines, ustekinumab was also the most used agent (Table 2A).

A total of 496 (7%), 128 (5%) and 32 (5%) CD patients started 1st, 2nd and 3rd LoT, respectively, for fistulising disease. In this setting, infliximab was the most used drug in 1st LoT in both bio-naïve patients and those previously exposed to a targeted therapy for another CD indication (Supplementary Table S3). Regarding prophylaxis of post-surgical recurrence, 5% of the CD patients in 1st and 2nd LoT, and 7% in 3rd LoT received targeted therapies for this indication (Supplementary Table S4).

#### 3.2.2. Patterns of Therapy Changes in Different Lines of Treatment

**Figure 3:**
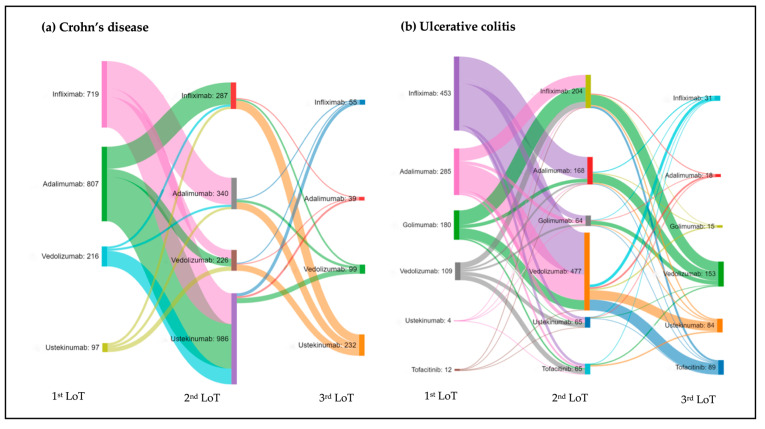
Treatment change distribution for Crohn’s disease (**a**) and ulcerative colitis (**b**) in different lines of treatment (LoT), including originator and biosimilars. Chart shows changes from 1st LoT to 2nd LoT, and from 2nd LoT to 3rd LoT. Values are provided as number of patients (n).

In CD, 71% of patients required one LoT, while 22% needed two LoT, and 7% three or more needed a different LoT (Table 1). Overall, in CD the main swap (i.e., change to a treatment with a different MoA) after discontinuation of a therapy was from anti-TNF to ustekinumab (36%). In 1st LoT, this change of therapy was more frequent from adalimumab (23%), and in 2nd LoT from infliximab (18%). The second most frequent treatment change in 1st LoT by MoA was a switch from one anti-TNF to another (25%), being the most common from infliximab to adalimumab (14%) (Figure 3a).

Among patients who had to discontinue both 1st and 2nd LoT, the most frequent reason was secondary LOR, which occurred in most cases with adalimumab, as it was the most used anti-TNF (48% and 30% in 1st and 2nd LoT, respectively), after a median treatment duration of 546 (518) and 487 (465) days in 1st and 2nd LoT, respectively. When the reason for 1st or 2nd LoT discontinuation was the onset of adverse events or PNR, the median treatment duration did not exceed the first year of treatment in both diseases (Supplementary Tables S5a and S6a).

The reason for 1st LoT discontinuation influenced the choice of the 2nd LoT. When the reason was PNR to infliximab or adalimumab, the most frequent change was a swap to ustekinumab, followed by the switch to a second anti-TNF. In contrast, following secondary LOR to one anti-TNF or after the onset of adverse events, treatment was switched to another anti-TNF in most cases (Supplementary Table S7a).

With respect to 3rd LoT, after failure of a second agent, the most frequent swap was from infliximab to ustekinumab (18%), followed by adalimumab to ustekinumab (16%), and vedolizumab to ustekinumab (13%). Only 4% of the patients changed from an anti-TNF to another anti-TNF agent (Figure 3a). In 3rd LoT, the main reason for treatment discontinuation was PNR in patients treated with vedolizumab and ustekinumab, with similar frequency (43%) (Supplementary Table S5a).

### 3.3. Ulcerative Colitis

#### 3.3.1. Patterns of Targeted Therapies by Line of Treatment

In UC, anti-TNF were the most widely used treatments in 1st LoT, and their use remained stable throughout time (85% in 2015 vs. 83% in 2021, *p* = 0.6) (Figure 2b). Among anti-TNF, infliximab was predominant throughout the study period, followed by adalimumab and golimumab (45% vs. 26% vs. 14%, *p* < 0.001, respectively). Overall, vedolizumab was the second most used drug by MoA as first-line (15%) (Table 2B).

In 2nd LoT, anti-TNF and vedolizumab were similarly used in general (45% vs. 44%), with a small proportion of patients receiving either ustekinumab or tofacitinib (6% in both cases) (Table 2B). Vedolizumab use increased progressively throughout the study period (22% in 2015 vs. 36% in 2021, *p* < 0.16), becoming the most prescribed drug in 2nd LoT since 2016 (Figure 2b).

In 3rd LoT, vedolizumab was the most used drug throughout the whole period (40%) (Table 2B), although its use decreased over time (92% in 2016 vs. 25% in 2021, *p* < 0.001). In contrast, the use of ustekinumab increased progressively (4% in 2017 vs. 30% in 2021, *p* < 0.001), becoming the main 3rd LoT in 2021, followed by vedolizumab and tofacitinib (30% vs. 25% vs. 24% in this year, respectively). In 4th LoT and subsequent lines, ustekinumab and tofacitinib were the most used therapies in 2021 (Supplementary Table S8).

#### 3.3.2. Patterns of Therapy Changes in Different Lines of Treatment

In UC, 62% of the patients required only one LoT, 23% had to receive two LoT, and 15% needed three or more different LoT, a higher percentage than that observed in CD (7%) (Table 1). Overall, the main swap after discontinuation of the first agent was from anti-TNF (mostly infliximab) to vedolizumab (42%). The second most frequent change was a switch from one anti-TNF to another anti-TNF (33%). In 2nd LoT, the most common swap was from infliximab to vedolizumab, and from vedolizumab to tofacitinib, with a similar frequency (16%) (Figure 3b).

Among patients who had to discontinue both 1st and 2nd LoT, the most frequent reason was secondary LOR, which occurred in most cases with infliximab in 1st LoT (37%) and with vedolizumab in 2nd LoT (49%), as they were the most commonly used drugs in 1st and 2nd LoT, respectively (Supplementary Table S5b). In 1st LoT, the median duration of treatment before secondary LOR was longer with vedolizumab than with infliximab or adalimumab (532 vs. 305 vs. 335 days, *p* < 0.001, respectively). The same situation was observed in 2nd LoT (Supplementary Table S6b).

The choice of the 2nd LoT depended on the reason for discontinuation of the 1st LoT. After either primary or secondary failure, the most common change was a swap to vedolizumab from infliximab or adalimumab in most patients, followed by a switch to a second anti-TNF. If anti-TNF was discontinued due to an onset of adverse events, the treatment was switched to another anti-TNF in most of the cases (Supplementary Table S7b).

In 3rd LoT, the main reason for treatment discontinuation was PNR, mostly in patients treated with vedolizumab (52%). In general, in both CD and UC, treatment discontinuation because of adverse events was associated mainly with infliximab (Supplementary Table S5).

### 3.4. Trends in Use of Biosimilars

**Figure 4:**
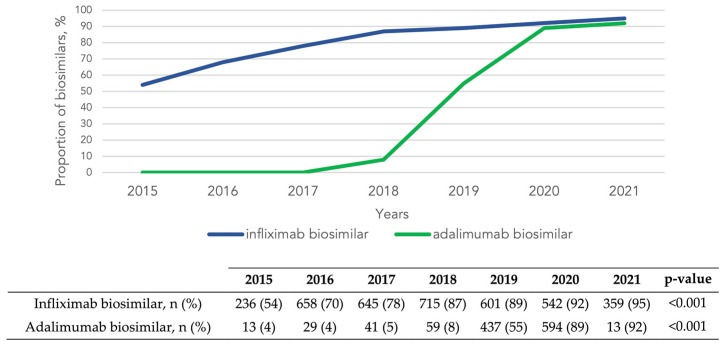
Trends in the use of biosimilars of each anti-TNF targeted therapy (infliximab and adalimumab) relative to their originals in inflammatory bowel disease from 2015 to 2021. Values of the graph are provided as %.

In both CD and UC, the use of biosimilars increased throughout the study period: the use of infliximab biosimilar increased from 54% in 2015 to 95% in 2021 (*p* < 0.001), and that of adalimumab biosimilar from 8% in 2018 to 92% in 2021 (*p* < 0.001) (Figure 4).

### 3.5. Factors Influencing Treatment Choice

In the Machine Learning analysis, several iterations were run for the prediction of different treatment patterns for each disease to get the best performing model in both 1st and 2nd LoT. However, we did not find any model that could adequately predict treatment choice; all of them showed suboptimal results in recall and precision metrics. The models with the best area under the curve are shown in Table 3. We found that the most important variables in these multiclass models were treatment availability, disease duration, and age at the time of prescription in 1st LoT; whereas in 2nd LoT, the most important variable was the previous targeted therapy.

## 4. Discussion

To our knowledge, this is one of the largest studies published in recent years on the usage of all targeted therapies approved to date in a nation-wide registry. Our main finding is that drug positioning seems to be different in CD and UC. In both diseases, anti-TNF plays a predominant role among targeted therapies, in which adalimumab is the most used drug in CD and infliximab in UC. However, the approval of new therapies in recent years has caused a decline in the use of these anti-TNF, especially from 2nd LoT onwards, resulting in the positioning of ustekinumab in CD and vedolizumab in UC as predominant second-line drugs. Indeed, we observed that 71% and 62% of the patients with CD and UC, respectively, required only one LoT.

The literature on trends in the use of targeted therapies to date is limited and has reported heterogeneous results. Brady et al. showed, in a cohort from the United States including patients from 2009 to 2013, that infliximab was the most commonly prescribed drug in 1st LoT in UC patients, while adalimumab was the most frequent in 1st LoT in CD patients, similar to our results [16]. By contrast, Jung et al. showed, in a Korean registry from 2010 to 2017, that infliximab was the most frequently used agent both in CD and UC [17]. These results are in agreement with a recent Danish cohort study including patients from 2015 to 2020, in which infliximab was the most prescribed biological agent in 1st LoT for both CD and UC, while adalimumab and vedolizumab were the main drugs in 2nd LoT in CD and UC, respectively [18]. However, these studies included patients only until 2020 and may not reflect the approval of adalimumab biosimilars and new therapies such as ustekinumab or tofacitinib.

Anti-TNF are still the most frequently prescribed drugs in 1st LoT. Because of the wide experience with their use in the past two decades, their good safety profile, their proven effectiveness in clinical practice, the availability of biosimilars, and reimbursement policies, these drugs are the first treatment choice in the majority of naïve patients with IBD [10]. In this respect, in Spain, vedolizumab, ustekinumab and tofacitinib are only reimbursed after failure of anti-TNF agents or in the case of contraindication for their use.

Our study showed that the most prescribed anti-TNF agents were adalimumab in CD and infliximab in UC. The reasons for this predominance are unclear because there are no studies comparing head-to-head the different anti-TNF with each other in IBD. In CD, the choice of adalimumab may be justified by patient’s preference due to the convenience of its subcutaneous formulations vs. the intravenous infusion of infliximab, although the influence on this choice of a relevant factor (physician’s preferences) could not be assessed. However, in fistulising CD, infliximab was the most prescribed treatment, in accordance with the available evidence; only infliximab was more effective than placebo in the closure of perianal fistulas in a randomized clinical trial [19], whereas evidence on the efficacy of adalimumab was provided by post hoc analyses with less conclusive results [20]. Nevertheless, both agents are good options and seem to be effective in this scenario. In luminal CD, several real-world studies showed similar effectiveness of infliximab and adalimumab [21]. Conversely, in UC the preference for infliximab could be influenced by clinicians’ general belief that infliximab is superior, or at least faster, than adalimumab. This is based on the results of pivotal randomized clinical trials comparing infliximab (ACT 1 and 2) or adalimumab (ULTRA 1 and 2) with placebo, in which the absolute difference in remission rates was lower for adalimumab than that shown by infliximab [22,23]. Indeed, indirect comparisons reported in network meta-analyses concluded that infliximab was superior to adalimumab for the induction of clinical remission in bio-naïve UC patients [24]. Finally, in patients with acute severe UC, infliximab is the only targeted therapy that has demonstrated superiority over placebo in a randomized clinical trial [25], whereas other biologics have not been evaluated in randomized trials in this scenario.

Another main finding of our study was the increasing trend to use ustekinumab in CD and vedolizumab in UC as 2nd LoT. In CD we found that, after its approval, ustekinumab displaced vedolizumab as the most used drug in 2nd LoT, despite the lack of comparative trials and the low-quality evidence from indirect studies. This clinicians’ preference may be based on the results of the GEMINI 3 trial, in which vedolizumab was not more effective than placebo in inducing clinical remission at week 6 in CD patients with previous anti-TNF failure, suggesting that the benefits of vedolizumab may not become evident until week 10 [26]. In addition, several real-world studies showed better results with ustekinumab compared to vedolizumab in anti-TNF refractory CD patients [27,28,29]. Regarding UC, vedolizumab progressively gained importance in 1st and 2nd LoT. Its increased use, at least in part, may have been influenced by the results of the VARSITY trial and by guideline recommendations of the American Gastroenterology Association, in which vedolizumab is considered superior to adalimumab for induction of remission in bio-naïve UC patients [7,30]. However, there is insufficient evidence to suggest the superiority of vedolizumab over other agents after anti-TNF failure. On one hand, in a recent network meta-analysis, tofacitinib and ustekinumab were ranked highest for inducing remission in UC patients with prior exposure to anti-TNF and they were considered more effective than vedolizumab or adalimumab [24]. On the other hand, Vickers et al. could not find differences at induction between vedolizumab and adalimumab in patients previously exposed to anti-TNF [31]. In our study, ustekinumab and tofacitinib were less frequently used than vedolizumab in 2nd LoT, probably because they were the most recently approved drugs for UC in our study period. However, since their use has increased in recent years, the trends observed in our study are likely to change in the coming years.

We observed that the reason for discontinuing the 1st LoT influenced the choice of the 2nd LoT. In routine clinical practice, a swap to a drug with a different MoA is common after primary failure to anti-TNF, while after secondary failure, switching to a second anti-TNF is a well-established option [10]. In our study we observed that in CD, following a PNR to anti-TNF, treatment was swapped to ustekinumab in most cases, whereas if the reason of failure was secondary LOR or onset of adverse events, treatment was mostly switched to a second anti-TNF. In UC the sequence was similar; vedolizumab was the most used drug after PNR to adalimumab or infliximab, whereas a second anti-TNF was preferentially used in case of adverse events. However, we found that secondary LOR to adalimumab or infliximab was also frequently followed by a swap to vedolizumab. This fact underlines the increasing importance of vedolizumab in UC.

In addition, we found that the second most frequent change of treatment in both CD and UC was a switch from an anti-TNF to a second anti-TNF agent, even in patients with primary failure, although this strategy has been shown to be less effective in this setting [10]. Nevertheless, Casanova et al. showed, in a retrospective study, that approximately half of the patients receiving a second anti-TNF after PNR to a prior drug achieved remission in the short term [32]. Therefore, our real-life results support that switching to another anti-TNF regardless of the reason for discontinuation of the first anti-TNF may be a reasonable option.

Despite an initial reticence to use biosimilars, these compounds have gained prominence, and mounting evidence supports their use in clinical practice, as well as the switch to their use in patients who start on the original drug [33,34]. Moreover, their introduction in IBD treatment is expected to reduce the economic burden on healthcare systems [35]. Our study provides real-world data on the remarkable increase in their use since their approval, showing that they accounted for more than 90% of all anti-TNF prescribed in 2021.

Our study has several limitations. Firstly, this is an observational study showing treatment patterns in recent years, but we did not evaluate whether it was the most appropriate positioning. Furthermore, neither did we assess the efficacy of each treatment in the different LoT or in terms of the reason for discontinuation of previous LoT, as these were not the aims of our study. Secondly, because the data was obtained from a prospectively maintained database, we did not have information on all the reasons for treatment initiation or discontinuation. Thirdly, we did not analyze the influence on the choice of each drug of some external factors such as clinicians’ preferences, their experience with the drug or even the price and availability of different therapies in the different hospitals. These variables are difficult to obtain and were not available in our study. Since treatment choice is probably largely influenced by these factors, their absence from our database may explain why we could not find, through Machine Learning tools, a model capable of accurately predicting specific treatments for individual patients. Finally, the Machine Learning models were applied to both 1st and 2nd LoT in a time period when not all drugs were initially available, which may have hampered the characterization of treatment patterns. Future studies should take into account all the aforementioned variables and apply the models in a scenario where all drugs are available throughout the study.

Nevertheless, our study has some strengths. Its sample size is large and therefore provides relevant data on the patterns of use of targeted therapies and their real-world positioning in recent years in Spain. In addition, this study also shows how the emergence of drugs with new therapeutic targets (vedolizumab, ustekinumab, tofacitinib) and the approval of biosimilar drugs have influenced the use of existing therapies.

In conclusion, drug positioning in IBD was heterogeneous and changed throughout the study period. Anti-TNF were the most used drugs in IBD and remained the preferred first-line targeted therapy in most patients, probably due, at least in part, to reimbursement policies. Among them, the most used agents were adalimumab in CD, and infliximab in UC. However, in 1st and 2nd LoT in CD, the use of anti-TNF agents decreased, while ustekinumab gained importance. In UC, anti-TNF prescription remained stable, with vedolizumab becoming the most used drug in 2nd LoT. The approval of biosimilars exerted a great impact on treatment, with a notable increase in their use. Machine Learning tools were unable to provide a model capable of predicting prescription patterns for these agents. To establish an ideal therapeutic algorithm for IBD, positioning studies evaluating the impact of new emerging therapies are needed in the near future, together with head-to-head comparative studies.

## Figures and Tables

**Table 1:** Baseline characteristics of the study population (N = 10,009).

	Crohn’s Disease (*n* = 7089)	Ulcerative Colitis (*n* = 2920)
Male gender, *n* (%)	3657 (52)	1580 (54)
Mean age at diagnosis (SD)	43 (16)	45 (16)
Extraintestinal manifestations, *n* (%)	1958 (29)	621 (22)
Smoking history, *n* (%) Smoker at diagnosis Ex-smoker	3393 (52) 2844 (44) 549 (8)	917 (35) 418 (16) 499 (19)
Family history, *n* (%)	1120 (17)	364 (14)
Surgery treatment ^a^, *n* (%) Abdominal Perianal Both	3120 (44) 2076 (66) 697 (22) 384 (12)	392 (13)
Montreal location at Crohn’s disease diagnosis, *n* (%)		
L1 (ileal) L2 (colonic) L3 (ileocolonic) L4 (upper gastrointestinal tract)	1899 (29) 936 (14) 2918 (45) 737 (11)	
Montreal behavior at Crohn’s disease diagnosis, *n* (%)		
B1 (inflammatory) B2 (stricturing) B3 (fistulising)	3933 (55) 1656 (23) 1500 (21)	
Perianal disease, *n* (%)	2128 (30)	
Ulcerative colitis extension, *n* (%)		
E1 (proctitis) E2 (left-sided colitis) E3 (extensive colitis)		189 (6) 1136 (39) 1576 (54)
Prior or concomitant use of immunomodulators, *n* (%)	5765 (81)	2254 (77)
Lines of treatment with targeted therapies, *n* (%)		
1st LoT 2nd LoT 3rd LoT or more ^b^	5014 (71) 1533 (22) 542 (7)	1820 (62) 681 (23) 418 (15)

SD, standard deviation; LoT, lines of treatment. ^a^ Total number of surgeries (patients could have had more than one). ^b^ Some patients received up to six lines of treatment.

**Table 2:** Prescription of targeted therapies for Crohn’s disease (**A**) and ulcerative colitis (**B**) in Spain from 2015 to 2021 according to line of treatment (LoT). N = 14,476 treatments.

A. Crohn’s Disease	1st LoT	2nd LoT	3rd LoT	4th LoT	5th LoT	6th LoT	Total
anti-TNFα, *n* (%)	5698 (80.5)	864 (41.5)	141 (26.1)	34 (28.1)	10 (38.5)	0 (0.0)	6747 (68.5)
Infliximab, *n* (%)	2527 (35.7)	431 (20.7)	81 (14.9)	24 (19.9)	6 (23.1)	0 (0.0)	3069 (31.2)
Infliximab originator	508 (7.2)	65 (3.1)	13 (2.4)	2 (1.7)	0 (0.0)	0 (0.0)	588 (6.0)
Infliximab biosimilar	2019 (28.5)	366 (17.6)	68 (12.5)	22 (18.2)	6 (23.1)	0 (0.0)	2481 (25.2)
Adalimumab, *n* (%)	3158 (44.6)	428 (20.6)	57 (10.6)	10 (8.2)	4 (15.4)	0 (0.0)	3657 (37.1)
Adalimumab originator	2026 (28.6)	293 (14.1)	28 (5.2)	5 (4.1)	2 (7.7)	0 (0.0)	2354 (23.9)
Adalimumab biosimilar	1132 (16.0)	135 (6.5)	29 (5.4)	5 (4.1)	2 (7.7)	0 (0.0)	1303 (13.2)
Vedolizumab, *n* (%)	520 (7.3)	236 (11.4)	110 (20.3)	33 (27.3)	7 (26.9)	1 (25.0)	907 (9.2)
Ustekinumab, *n* (%)	868 (12.2)	972 (46.8)	287 (53.0)	54 (44.6)	8 (30.8)	3 (75.0)	2192 (22.2)
Overall, *n*	7089	2075	542	121	26	4	9857
**B. Ulcerative Colitis**	**1st LoT**	**2nd LoT**	**3rd LoT**	**4th LoT**	**5th LoT**	**6th LoT**	**Total**
anti-TNFα, *n* (%)	2453 (83.9)	492 (44.8)	77 (18.4)	18 (13.9)	7 (17.5)	1 (11.1)	3048 (66.1)
Infliximab, *n* (%)	1304 (44.6)	246 (22.4)	41 (9.8)	12 (9.3)	4 (10.0)	0 (0.0)	1607 (34.8)
Infliximab originator	264 (9.0)	49 (4.5)	10 (2.4)	1 (0.8)	1 (2.5)	0 (0.0)	325 (7.0)
Infliximab biosimilar	1040 (35.6)	197 (17.9)	31 (7.4)	11 (8.5)	3 (7.5)	0 (0.0)	1282 (27.8)
Adalimumab, *n* (%)	747 (25.5)	179 (16.3)	21 (5.0)	3 (2.3)	3 (7.5)	0 (0.0)	953 (20.7)
Adalimumab originator	486 (16.6)	119 (10.8)	11 (2.6)	2 (1.5)	0 (0.0)	0 (0.0)	618 (13.4)
Adalimumab biosimilar	261 (8.9)	60 (5.5)	10 (2.4)	1 (0.8)	3 (7.5)	0 (0.0)	335 (7.3)
Golimumab, *n* (%)	402 (13.8)	67 (6.1)	15 (3.6)	3 (2.3)	0 (0.0)	1 (11.1)	488 (10.6)
Vedolizumab, *n* (%)	431 (14.8)	479 (43.5)	165 (39.4)	10 (7.7)	4 (10.0)	1 (11.1)	1090 (23.6)
Ustekinumab, *n* (%)	22 (0.8)	65 (5.9)	84 (20.0)	47 (36.2)	17 (42.5)	2 (22.2)	238 (5.2)
Tofacitinib, *n* (%)	14 (0.5)	64 (5.8)	93 (22.2)	55 (42.3)	12 (30.0)	5 (55.6)	243 (5.3)
Overall, *n*	2920	1100	419	130	40	9	4619

**Table 3:** Machine Learning models. Results of 4th and 1st iterations in 1st and 2nd line of treatment (LoT), respectively, in Crohn’s disease (**A**). Results of 3rd and 1st iterations in 1st and 2nd LoT, respectively, in ulcerative colitis (**B**). Table includes target treatments to be predicted, all the variables considered and results.

A. Crohn’s Disease				
1st LoT. 4th iteration.				
Target	Variables	Results		
Adalimumab Infliximab Vedolizumab Ustekinumab	Demographic variables Clinical variables Treatment variables Hospitals Surgeries	Label	recall	precision
		Adalimumab	0.38	0.58
		Infliximab	0.40	0.53
		Vedolizumab	0.46	0.19
		Ustekinumab	0.62	0.30
		*Logistic regression*		
2nd LoT. 1st iteration.				
Adalimumab biosimilar Adalimumab originator Infliximab biosimilar Infliximab originator Ustekinumab Vedolizumab	Demographic variables Clinical variables Treatment variables Hospitals Surgeries	Label	recall	precision
		Adalimumab_biosim	0.79	0.43
		Adalimumab_orig	0.76	0.39
		Infliximab_biosim	0.21	0.23
		Infliximab_original	0.41	0.16
		Ustekinumab	0.46	0.72
		Vedolizumab	0.21	0.54
		*Random Forest model*		
B. Ulcerative Colitis				
1st LoT, 3rd iteration.				
Adalimumab Infliximab Other original ^a^ Vedolizumab	Demographic variables Clinical variables Treatment variables Hospitals Surgeries	Label	recall	precision
		Adalimumab	0.41	0.37
		Infliximab	0.50	0.61
		Other_orig	0.35	0.28
		Vedolizumab	0.34	0.30
		*XGBoost*		
2nd LoT, 1st iteration.				
Adalimumab biosimilar Adalimumab original Infliximab biosimilar Infliximab original Golimumab Tofacitinib Vedolizumab	Demographic variables Clinical variables Treatment variables Hospitals Surgeries	Label	recall	precision
		Adalimumab_biosim	0.65	0.28
		Adalimumab_orig	0.74	0.29
		Golimumab	0.29	0.14
		Infliximab_biosim	0.22	0.33
		Infliximab_original	0.47	0.19
		Tofacitinib	1.00	0.97
		Ustekinumab	0.53	0.19
		Vedolizumab	0.00	0.33
		*Random Forest model*		

^a^ Other original targets included golimumab, ustekinumab and tofacitinib.

## Data Availability

The data underlying this article will be shared on reasonable request to the corresponding author.

## Supplementary Materials

The following supporting information can be downloaded at: https://www.mdpi.com/article/10.3390/pharmaceutics16050629/s1, Table S1: Treatment distribution per healthcare center score (IASIST level) and by line of treatment (LoT) in Crohn’s disease (A) and ulcerative colitis (B); Table S2: Prescription of targeted therapies for inflammatory bowel disease in Spain since 2015; Table S3: Treatment patterns in patients who received targeted therapies for fistulizing Crohn’s disease; Table S4: Treatment indications in Crohn’s disease and ulcerative colitis by line of treatment; Table S5: Reasons for treatment discontinuation in Crohn’s disease (A) and ulcerative colitis (B) by line of treatment in from 2015 to 2021; Table S6: Duration of treatments in days by reasons for discontinuation and by line of treatment in Crohn’s disease (A) and in ulcerative colitis (B); Table S7: targeted therapy chosen in 2nd line of treatment (LoT) depending on the reason for discontinuation of the 1st LoT; Table S8: Prescription of targeted therapies for ulcerative colitis in 2016 (A) and 2021 (B) by line of treatment.
