# Supplementary material for: Trends in Targeted Therapy Usage in Inflammatory Bowel Disease: TRENDY Study of ENEIDA
Source: Pharmaceutics. 2024 May 8;16(5):629. doi: 10.3390/pharmaceutics16050629 (PMC11124941; doi:10.3390/pharmaceutics16050629)
Supplement: Supplementary file 1 [file pharmaceutics-16-00629-s001.zip › pharmaceutics-2948309-supplementary.pdf]

SUPPLEMENTARY MATERIALS

SUPPLEMENTARY TABLE S1: Treatment distribution per healthcare centre score (IASIST level) and by line of treatment (LoT) in Crohn's disease (A) and ulcerative colitis (B).

Crohn's disease

A	IASIST level	**Anti-TNFα**, *n* (%)	Infliximab, *n* (%)	Infliximab originator	Infliximab biosimilar	Adalimumab, *n* (%)	Adalimumab originator	Adalimumab biosimilar	**Vedolizumab**, *n* (%)	**Ustekinumab**, *n* (%)	Overall, *n*
1st Lot	2	61 (87.1)	36 (51.4)	12 (17.1)	24 (34.3)	25 (35.7)	22 (31.4)	3 (4.3)	3 (4.3)	6 (8.6)	70
	3	672 (84.6)	245 (30.8)	67 (8.4)	178 (22.4)	426 (53.7)	281 (35.4)	145 (18.3)	50 (6.3)	71 (9)	792
	4	1,511 (80)	693 (36.7)	94 (5)	599 (31.7)	814 (43.1)	500 (26.5)	314 (16.6)	149 (7.9)	227 (12)	1,883
	5	3,159 (78.9)	1,434 (35.8)	310 (7.7)	1,124 (28.1)	1,717 (42.9)	1,107 (27.7)	610 (15.2)	302 (7.5)	539 (13.5)	3,992
2nd LoT	2	2 (12.4)	1 (6.2)	0 (0)	1 (6.2)	1 (6.2)	1 (6.2)	0 (0)	2 (12.5)	12 (75.0)	16
	3	105 (45)	53 (22.7)	11 (4.7)	42 (18)	51 (21.9)	40 (17.2)	11 (4.7)	19 (8.2)	109 (46.8)	232
	4	211 (40.2)	95 (18.1)	11 (2.1)	84 (16)	114 (21.7)	71 (13.5)	43 (8.2)	75 (14.3)	239 (45.5)	523
	5	507 (41.4)	261 (21.3)	40 (3.3)	221 (18)	244 (19.9)	168 (13.7)	76 (6.2)	136 (11.1)	580 (47.3)	1,221
3rd LoT	2	0 (0)	0 (0)	0 (0)	0 (0)	0 (0)	0 (0)	0 (0)	0 (0)	2 (100)	2
	3	10 (13.3)	10 (13.3)	3 (4)	7 (9.3)	0 (0)	0 (0)	0 (0)	16 (21.3)	49 (65.3)	75
	4	34 (24.1)	17 (12)	2 (1.4)	15 (10.6)	16 (11.4)	9 (6)	7 (5)	36 (25.5)	71 (50.4)	140
	5	91 (29.8)	49 (16)	5 (1.6)	44 (14.4)	40 (13.1)	19 (6.2)	21 (6.9)	56 (18.3)	155 (50.7)	300

Ulcerative colitis

**B**	IASIST level	**Anti-TNFα**, *n* (%)	Infliximab, *n* (%)	Infliximab originator	Infliximab biosimilar	Adalimumab, *n* (%)	Adalimumab originator	Adalimumab biosimilar	Golimumab, *n* (%)	**Vedolizumab**, *n* (%)	**Ustekinumab**, *n* (%)	**Tofacitinib**, *n* (%)	Overall, *n*
1st LoT	2	10 (71.4)	7 (50)	1 (7.1)	6 (42.9)	2 (14.3)	2 (14.3)	0 (0)	1 (7.1)	4 (28.6)	0 (0)	0 (0)	14
	3	284 (89.3)	165 (51.9)	34 (10.7)	131 (41.2)	92 (28.9)	55 (17.3)	37 (11.6)	27 (8.5)	29 (9.1)	3 (0.9)	2 (0.6)	318
	4	657 (85.1)	359 (46.5)	70 (9.1)	289 (37.4)	194 (25.1)	110 (14.2)	84 (10.9)	104 (13.5)	107 (13.8)	5 (0.6)	4 (0.5)	773
	5	1,372 (82.5)	705 (42.4)	155 (9.3)	550 (33.1)	423 (25.4)	297 (17.8)	126 (7.6)	244 (14.7)	273 (16.4)	11 (0.7)	8 (0.5)	1,664
2nd LoT	2	2 (50)	2 (50)	2 (50)	0 (0)	0 (0)	0 (0)	0 (0)	0 (0)	1 (25)	1 (25)	0 (0)	4
	3	52 (44.8)	24 (20.7)	6 (5.2)	18 (15.5)	22 (18.9)	12 (10.3)	10 (8.6)	6 (5.2)	42 (36.2)	10 (8.6)	12 (10.3)	116
	4	123 (41.7)	55 (18.6)	13 (4.4)	42 (14.2)	43 (14.6)	28 (9.5)	15 (5.1)	25 (8.5)	153 (51.9)	13 (4.4)	6 (2)	295
	5	285 (45.4)	149 (23.8)	28 (4.5)	121 (19.3)	105 (16.7)	74 (11.8)	31 (4.9)	31 (4.9)	266 (42.4)	36 (5.7)	40 (6.4)	627
3rd LoT	2	0 (0)	0 (0)	0 (0)	0 (0)	0 (0)	0 (0)	0 (0)	0 (0)	0 (0)	0 (0)	0 (0)	1
	3	7 (18.9)	3 (8.1)	1 (2.7)	2 (5.4)	2 (5.4)	0 (0)	2 (5.4)	2 (5.4)	12 (32.4)	7 (18.9)	11 (29.7)	37
	4	20 (18.7)	9 (8.4)	1 (0.9)	8 (7.5)	4 (3.7)	1 (0.9)	3 (2.8)	7 (6.6)	44 (41.5)	27 (25.5)	15 (14.2)	106
	5	47 (18.4)	27 (10.5)	8 (3.1)	19 (7.4)	15 (5.9)	10 (3.9)	5 (2)	5 (2.0)	97 (37.9)	46 (18)	66 (25.8)	256

SUPPLEMENTARY TABLE S2. Prescription of targeted therapies for inflammatory bowel disease in Spain since 2015. n=14,463.

	2015	2016	2017	2018	2019	2020	2021	p value
**anti-TNFα**, *n (%)*	828 (87.1)	1,805 (81.7)	1,704 (72.6)	1,685 (62.1)	1,558 (60.6)	1,301 (60.1)	907 (60.2)	<0.001
Infliximab, *n (%)*	436 (45.8)	944 (42.7)	829 (35.3)	821 (30.3)	672 (26.1)	589 (27.2)	378 (25.1)	-
Infliximab originator	200 (21)	286 (12.9)	184 (7.8)	106 (3.9)	71 (2.8)	47 (2.2)	19 (1.3)	-
Infliximab biosimilar	236 (24.8)	658 (29.8)	645 (27.5)	715 (26.3)	601 (23.4)	542 (25)	359 (23.8)	-
Adalimumab, *n (%)*	358 (37.6)	782 (35.4)	752 (32)	757 (27.9)	790 (30.7)	666 (30.8)	505 (33.5)	-
Adalimumab originator	345 (36.3)	753 (34.1)	711 (30.3)	698 (25.7)	353 (13.7)	72 (3.3)	40 (2.7)	-
Adalimumab biosimilar	13 (1.4)	29 (1.3)	41 (1.7)	59 (2.2)	437 (17)	594 (27.4)	465 (30.9)	-
Golimumab, *n (%)*	34 (3.6)	79 (3.6)	123 (5.2)	107 (3.9)	96 (3.7)	46 (2.1)	24 (1.6)	-
**Vedolizumab**, *n (%)*	106 (11.1)	354 (16)	354 (15.1)	371 (13.7)	373 (14.5)	269 (12.4)	170 (11.3)	0.07
**Ustekinumab**, *n (%)*	16 (1.7)	51 (2.3)	280 (11.9)	632 (23.3)	581 (22.6)	497 (23)	373 (24.8)	<0.001
**Tofacitinib**, *n (%)*	1 (0.1)	0 (0.0)	10 (0.4)	26 (1)	58 (2.3)	97 (4.5)	56 (3.7)	<0.001
Overall, *n*	951	2,210	2,348	2,714	2,570	2,164	1,506	-

**SUPPLEMENTARY TABLE S3:** Treatment patterns in patients who received targeted therapies for fistulizing Crohn's disease. n=229 patients. Line of treatment (LoT).							
**Fistulizing Crohn's disease**							
**Bio-naïve**							
	Anti-TNF, *n (%)*	Infliximab, *n (%)*	Adalimumab, *n (%)*	Ustekinumab, *n (%)*	Vedolizumab, *n (%)*	Total, *n*	P value
1st LoT	160 (84.7)	99 (52.3)	61 (32.2)	16 (8.4)	13 (6.8)	189	<0.001
2nd LoT	12 (57.1)	7 (33.3)	5 (23.8)	8 (38)	1 (4.7)	21	0.14
3rd LoT	2 (100)	1 (50)	1 (50)	0 (0)	0 (0)	2	0.57
**Prior use of biologics for CD treatment**							
1 biologic							
1st LoT	21 (67.8)	15 (48.3)	6 (19.3)	7 (22.5)	3 (9.6)	31	0.01
2nd LoT	2 (50)	1 (25)	1 (25)	2 (50)	0 (0)	4	0.57
2 biologics							
1st LoT	4 (50)	3 (37.5)	1 (12.5)	3 (37.5)	1 (12.5)	8	0.57

**SUPPLEMENTARY TABLE S4:** Treatment indications in Crohn's disease and ulcerative colitis by line of treatment (LoT).							
		Crohn's disease			Ulcerative colitis		
		1st LoT	2nd LoT	3rd LoT	1st LoT	2nd LoT	3rd LoT
Treatment indication	Induction of remission, *n (%)*	5,368 (76)	1,750 (74)	520 (77)	2,577 (88)	1,012 (88)	353 (92)
	Maintenance of remission, *n (%)*	637 (9)	265 (11)	52 (8)	211 (7)	96 (8)	24 (6)
	Fistulizing disease, *n (%)*	497 (7)	128 (5)	32 (5)	-	-	-
	Prophylaxis of post-surgical recurrence, *n (%)*	333 (5)	128 (5)	51 (7)	-	-	-
	Other, *n (%)*	254 (4)	96 (4)	21 (3)	132 (5)	46 (4)	8 (2)
	Total	7,089	2,368	676	2,920	1,154	385

SUPPLEMENTARY TABLE S5: Reasons for treatment discontinuation in Crohn's disease (A) and ulcerative colitis (B) by line of treatment (LoT) in from 2015 to 2021.

A. Crohn's disease.

Reasons for treatment discontinuation		Anti-TNFα	Infliximab	Adalimumab	Vedolizumab	Ustekinumab	Overall, *n*
1st LoT	Adverse event, *n (%)*	347 (89.4)	184 (47.4)	163 (42)	25 (6.4)	16 (4.1)	388
	Primary non-response, *n (%)*	303 (69.3)	132 (30.2)	168 (38.4)	75 (17.2)	59 (13.5)	434
	Loss of secondary response, *n (%)*	697 (83)	290 (34.5)	406 (48.4)	91 (10.8)	51 (6.1)	838
	Sustained remission, *n (%)*	101 (100)	70 (69.3)	31 (30.7)	0 (0)	0 (0)	101
	Other, *n (%)*	601 (86.9)	351 (50.8)	249 (36)	49 (7.1)	41 (5.9)	690
2nd LoT	Adverse event, *n (%)*	65 (73.8)	41 (46.6)	24 (27.2)	7 (8.0)	16 (18.2)	88
	Primary non-response, *n (%)*	61 (33.8)	34 (18.8)	26 (14.4)	41 (22.7)	79 (43.6)	180
	Loss of secondary response, *n (%)*	119 (56.1)	54 (25.5)	64 (30.1)	46 (21.7)	47 (22.2)	211
	Sustained remission, *n (%)*	8 (80.0)	5 (50.0)	3 (30)	1 (10)	1 (10)	10
	Other, *n (%)*	84 (61.2)	53 (38.6)	31 (22.6)	16 (11.7)	37 (27)	137
3rd LoT	Adverse event, *n (%)*	16 (80)	12 (60.0)	4 (20)	2 (10)	2 (10)	20
	Primary non-response, *n (%)*	9 (14.4)	4 (6.4)	5 (8)	27 (42.9)	27 (42.9)	63
	Loss of secondary response, *n (%)*	14 (29.2)	7 (14.6)	7 (14.6)	11 (22.9)	23 (47.9)	48
	Sustained remission, *n (%)*	0 (0)	0 (0.0)	0 (0)	0 (0)	0 (0)	1
	Other, *n (%)*	7 (33.4)	6 (28.6)	1 (4.8)	2 (9.5)	12 (57.1)	21

B. Ulcerative colitis

Reasons for treatment discontinuation		Anti-TNFα	Infliximab	Adalimumab	Golimumab	Vedolizumab	Ustekinumab	Tofacitinib	Overall, n
1st LoT	Adverse event, *n (%)*	147 (92.4)	106 (66.7)	29 (18.2)	12 (7.5)	10 (6.3)	2 (1.3)	0 (0)	159
	Primary non-response, *n (%)*	300 (85.5)	126 (35.9)	106 (30.2)	68 (19.4)	51 (14.5)	0 (0)	0 (0)	351
	Loss of secondary response, *n (%)*	369 (85.3)	160 (37)	129 (29.8)	80 (18.5)	63 (14.5)	1 (0.2)	0 (0)	433
	Sustained remission, *n (%)*	68 (97.1)	52 (74.3)	12 (17.1)	4 (5.7)	2 (2.9)	0 (0)	0 (0)	70
	Other, *n (%)*	242 (85.5)	150 (53)	61 (21.5)	31 (11)	39 (13.8)	2 (0.7)	0 (0)	283

2nd LoT	Adverse event, *n (%)*	35 (83.3)	19 (45.2)	15 (35.7)	1 (2.4)	7 (16.7)	0 (0)	0 (0)	42
	Primary non-response, *n (%)*	89 (48.8)	42 (23)	32 (17.6)	15 (8.2)	87 (47.8)	6 (3.3)	0 (0)	182
	Loss of secondary response, *n (%)*	73 (49.7)	27 (18.4)	29 (19.7)	17 (11.6)	71 (48.3)	3 (2)	0 (0)	147
	Sustained remission, *n (%)*	8 (66.6)	4 (33.3)	3 (25)	1 (8.3)	4 (33.3)	0 (0)	0 (0)	12
	Other, *n (%)*	49 (71)	30 (43.5)	13 (18.8)	6 (8.7)	18 (26.1)	2 (2.9)	0 (0)	69
3rd LoT	Adverse event, *n (%)*	5 (71.4)	5 (71.4)	0 (0)	0 (0)	1 (14.3)	1 (14.3)	0 (0)	7
	Primary non-response, *n (%)*	13 (24.2)	7 (13)	4 (7.5)	2 (3.7)	28 (51.9)	13 (24.1)	0 (0)	54
	Loss of secondary response, *n (%)*	10 (20.3)	3 (6.1)	4 (8.1)	3 (6.1)	29 (59.2)	10 (20.4)	0 (0)	49
	Sustained remission, *n (%)*	0 (0)	0 (0)	0 (0)	0 (0)	1 (100)	0 (0)	0 (0)	1
	Other, *n (%)*	5 (38.5)	1 (7.7)	1 (7.7)	3 (23.1)	6 (46.2)	2 (15.4)	0 (0)	13

SUPPLEMENTARY TABLE S6: Duration of treatments in days by reasons for discontinuation and by line of treatment (LoT) in Crohn's disease (A) and in ulcerative colitis (B). Interquartile range (IQR); Non available (N/A).

A. **Crohn's disease**

			Infliximab	Adalimumab	Ustekinumab	Vedolizumab	p value
1st LoT	Adverse event	Median (IQR)	153 (334)	243 (379)	242 (442)	107 (266)	0.05
	Primary non-response		123 (213)	123 (173)	151 (197)	181 (181)	0.59
	Loss of secondary response		488 (518)	546 (518)	487 (441)	547 (397)	0.72
	Sustained remission		730 (608)	396 (366)	0 (0)	1156 (N/A)	0.01
	Other		335 (503)	426 (573)	337 (305)	274 (307)	0.001
2nd LoT	Adverse event	Median (IQR)	123 (381)	213 (267)	181 (227)	243 (412)	0.20
	Primary non-response		100 (151)	181 (273)	181 (243)	212 (246)	0.04
	Loss of secondary response		503 (586)	487 (465)	454 (426)	563 (402)	0.90
	Sustained remission		579 (245)	499 (229)	458 (N/A)	546 (N/A)	0.68
	Other		365 (532)	380 (738)	258 (397)	245 (614)	0.47

**B. Ulcerative colitis**									
			Infliximab	Adalimumab	Golimumab	Ustekinumab	Vedolizumab	Tofacitinib	P value
1st LoT	Adverse event	Median (IQR)	121 (334)	183 (306)	168 (272)	62 (N/A)	275 (303)	N/A	0.18
	Primary non-response		62 (93)	122 (120)	120 (92)	92 (60)	123 (152)	N/A	<0.001
	Loss of secondary response		305 (278)	335 (571)	271 (365)	396 (357)	532 (577)	N/A	<0.001
	Sustained remission		700 (446)	472 (380)	547 (212)	0 (0)	852 (388)	N/A	0.84
	Other		274 (431)	244 (471)	124 (233)	122 (N/A)	247 (429)	N/A	0.09
2nd LoT	Adverse event	Median (IQR)	227 (266)	243 (410)	274 (395)	184 (0)	107 (405)	N/A	0.98
	Primary non-response		120 (130)	122 (136)	120 (39)	123 (153)	122 (144)	N/A	0.83
	Loss of secondary response		244 (274)	305 (252)	411 (470)	517 (426)	411 (441)	N/A	0.004
	Sustained remission		883 (670)	791 (190)	806 (76)	0 (0)	487 (319)	N/A	0.19
	Other		273 (519)	160 (745)	77 (81)	365 (260)	214 (152)	N/A	0.02

**SUPPLEMENTARY TABLE S7:** Targeted therapy chosen in 2nd line of treatment (LoT) depending on the reason for discontinuation of the 1st LoT. Values are provided as number of treatments (n).				
**A. Crohn's disease**				
	Primary non-response	Loss of secondary response	Adverse event	Sustained remission
Infliximab	**Ustekinumab (25)**<br>Vedolizumab (11)<br>Adalimumab (14)	**Adalimumab (27)**<br>Vedolizumab (12)<br>Ustekinumab (10)	**Adalimumab (13)**<br>Ustekinumab (4)<br>Vedolizumab (2)	**Infliximab (8)**<br>Adalimumab (4)<br>Ustekinumab (1)
Adalimumab	**Ustekinumab (40)**<br>Infliximab (28)<br>Vedolizumab (13)	**Infliximab (34)**<br>Ustekinumab (24)<br>Vedolizumab (17)	**Infliximab (29)**<br>Ustekinumab (8)<br>Vedolizumab (5)	**Adalimumab (4)**<br>**Ustekinumab (4)**
**B. Ulcerative colitis**				
Infliximab	**Vedolizumab (48)**<br>Adalimumab (19)<br>Golimumab (5)<br>Ustekinumab (1)	**Vedolizumab (31)**<br>Adalimumab (16)<br>Golimumab (9)<br>Ustekinumab (1)	**Adalimumab (7)**<br>Vedolizumab (6)	**Infliximab (5)**<br>Adalimumab (3)<br>Vedolizumab (2)<br>Ustekinumab (1)
Adalimumab	**Vedolizumab (21)**<br>Infliximab (19)<br>Golimumab (4)<br>Ustekinumab (3)	**Vedolizumab (20)**<br>Infliximab (6)<br>Golimumab (3)	**Infliximab (5)**<br>Golimumab (1)<br>Vedolizumab (1)	
Golimumab	**Infliximab (18)**<br>Vedolizumab (8)<br>Adalimumab (6)	**Infliximab (15)**<br>Vedolizumab (7)<br>Adalimumab (4)	**Infliximab (7)**<br>Adalimumab (3)	**Golimumab (2)**

SUPPLEMENTARY TABLE S8: Prescription of targeted therapies for ulcerative colitis in 2016 (A) and 2021 (B) by line of treatment (LoT)

A		1st LoT	2nd LoT	3rd LoT	4th LoT	5th LoT	6th LoT	Total
2016	anti-TNFα, *n (%)*	447 (86.2)	78 (55)	1 (8.3)	0 (0)	0 (0)	0 (0.)	526 (78)
	Infliximab, *n (%)*	253 (48.8)	33 (23.3)	1 (8.3)	0 (0)	0 (0)	0 (0)	287 (42.6)
	Adalimumab, *n (%)*	131 (25.3)	31 (21.8)	0 (0)	0 (0)	0 (0)	0 (0)	162 (24)
	Golimumab, *n (%)*	63 (12.1)	14 (9.9)	0 (0)	0 (0)	0 (0)	0 (0)	77 (11.4)
	Vedolizumab, *n (%)*	71 (13.7)	64 (45.1)	11 (91.7)	0 (0)	0 (0)	0 (0)	146 (21.7)
	Ustekinumab, *n (%)*	1 (0.2)	0 (0)	0 (0)	0 (0)	0 (0)	0 (0)	1 (0.1)
	Tofacitinib, *n (%)*	0 (0)	0 (0)	0 (0)	0 (0)	0 (0)	0 (0)	0 (0)
	Overall, *n*	519	142	12	0	0	0	673
**B**		1st LoT	2nd LoT	3rd LoT	4th LoT	5th LoT	6th LoT	Total
2021	anti-TNFα, *n (%)*	222 (83.1)	59 (38.7)	16 (20.9)	5 (15.1)	1 (10)	1 (33.3)	304 (56.1)
	Infliximab, *n (%)*	116 (43.4)	32 (21)	10 (13.1)	2 (6.1)	1 (10)	0 (0)	161 (29.7)
	Adalimumab, *n (%)*	89 (33.3)	23 (15.1)	5 (6.5)	2 (6)	0 (0)	0 (0)	119 (22)
	Golimumab, *n (%)*	17 (6.4)	4 (2.6)	1 (1.3)	1 (3)	0 (0)	1 (33.3)	24 (4.4)
	Vedolizumab, *n (%)*	35 (13.1)	55 (36.2)	19 (25)	1 (3)	1 (10)	0 (0)	111 (20.5)
	Ustekinumab, *n (%)*	8 (3)	21 (13.8)	23 (30.3)	13 (39.4)	5 (50)	1 (33.3)	71 (13.1)
	Tofacitinib, *n (%)*	2 (0.7)	17 (11.2)	18 (23.7)	14 (42.4)	3 (30)	1 (33.3)	55 (10.2)
	Overall, *n*	267	152	76	33	10	3	541
